# International Normalized Ratio (INR) Increases amongst Two Patients Living with HIV on Warfarin after Being Switched from a Nevirapine to a Dolutegravir-Based Antiretroviral Regimen

**DOI:** 10.1155/2021/9384663

**Published:** 2021-10-29

**Authors:** Natalie Sang, Sonak Pastakia, Samuel Nyanje

**Affiliations:** ^1^Department of Pharmacology and Toxicology, Moi University School of Medicine, Eldoret, Kenya; ^2^Purdue University, College of Pharmacy, Indianapolis, IN, USA; ^3^Academic Model for Providing Access to Healthcare, Eldoret, Kenya

## Abstract

The increased use of dolutegravir-based regimens in the treatment of HIV is unmasking drug interactions, particularly in patients who were previously on nevirapine. Nevirapine is an enzyme inducer and increases the dosing requirements for cytochrome P450 enzyme substrates including warfarin. Upon discontinuing nevirapine, close monitoring of drugs with narrow therapeutic indices is paramount since dosing requirements may significantly reduce, increasing the probability of toxicity development. We present two cases describing interactions experienced by patients living with HIV, while transitioning from nevirapine to dolutegravir-based HIV regimens. The first case describes a 70-year-old man living with HIV and diabetes, while the second case describes a 60-year-old woman living with HIV. They were diagnosed with unprovoked deep vein thrombi, and while receiving treatment with warfarin, their HIV medication regimen was changed from lamivudine, zidovudine, nevirapine, and septrin to lamivudine, tenofovir, dolutegravir, and septrin. During the weeks following this switch, warfarin requirements decreased resulting in supratherapeutic INRs. With the continued promotion of dolutegravir-based HIV regimens as the preferred option for the treatment of HIV in President's Emergency Plan for AIDS Relief (PEPFAR) supported HIV treatment programs in Africa, clinicians must be aware of the potentially life-threatening consequences of switching antiretroviral regimens. It is hoped that a greater awareness of this potential side effect could lead to increased monitoring and prevention of the consequences of drug interactions.

## 1. Background

HIV is a well-described prothrombotic condition and is often complicated by venous thromboembolism (VTE) [1–3]. Several HIV specific factors including lower CD4 count, higher viral loads, and HIV-induced activation of TNF-*α*, D dimer, and IL-6 elevation may increase the risk of VTEs [2–4]. VTEs are treated with anticoagulants including warfarin and rivaroxaban for 3–6 months. Longer periods of anticoagulation may be required among people living with HIV (PLWH), hence increasing chances of drug interactions [5].

Older HIV regimens usually comprise of two nonnucleotide reverse transcriptase inhibitors (NRTIs), tenofovir and lamivudine or emtricitabine, and a nonnucleotide reverse transcriptase inhibitor, e.g., nevirapine and efavirenz, or a boosted protease inhibitor, e.g., lopinavir/ritonavir. Integrase strand transfer inhibitors (INSTIs), e.g., dolutegravir, were relatively recently approved for use in HIV treatment.

Due to the high barrier to resistance, ease of use, and improved tolerability, PEPFAR supported programs across Africa have introduced dolutegravir-based regimens as the preferred treatment option for HIV. The Kenyan National AIDS and Sexually Transmitted Infection Control Program (NASCOP) has also adopted these guidelines [6]. This shift has necessitated the subsequent phase out of older regimens, including nevirapine-based regimens, even in virologically suppressed patients. There are limited data on the impact of these switches, especially in the presence of other comorbidities and potentially interacting medications such as warfarin [7, 8].

Nevirapine is an enzyme inducer and enhances the hepatic metabolism of hepatic enzyme substrates including warfarin. There have been several prior reports of the interaction observed with the concomitant use of warfarin and nevirapine. Some patients required higher doses of warfarin when nevirapine was started, while others failed to achieve therapeutic INR until nevirapine was stopped [9]. One case study describes a patient who had been taking nevirapine and warfarin concomitantly for 8 months and experienced supratherapeutic INR when nevirapine was stopped [10]. With the unpredictability of drug interactions between nevirapine and warfarin, empiric dose adjustments are not typically recommended [11].

To the best of our knowledge, there are no published case reports of supratherapeutic INRs resulting from switching from a nevirapine-based regimen to a dolutegravir-based antiretroviral regimen amongst PLWH on warfarin.

## 2. Case Presentation

### 2.1. Case 1

**Figure 1:**
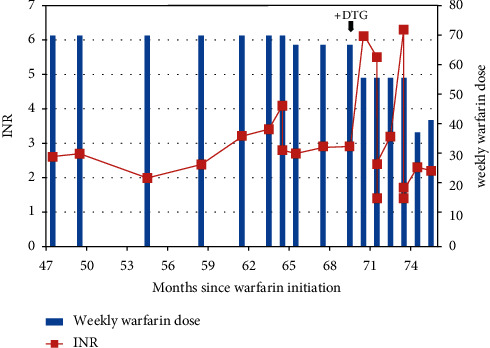
Graphical representation of case 1's INR and corresponding weekly warfarin dose during clinical visits.

The first patient is a 70-year-old man, diagnosed with HIV in 2004 and started on stavudine, lamivudine, and nevirapine in 2007. In September 2009, his antiretroviral regimen was switched to lamivudine, zidovudine, and nevirapine as stavudine was phased out of the treatments available from the national program. His viral load in February 2018 was 1168 copies/ml on this regimen. In March 2013, he was diagnosed with an unprovoked DVT with HIV being his only notable risk factor and started on warfarin with the aim of achieving an INR of 2-3. A therapeutic INR of 2.7 was achieved in September 2014 at a warfarin dose of 63 mg per week (9 mg once daily). Two lower limb Doppler ultrasounds completed 6 months and 1 year after initiation of warfarin showed chronic DVT and led to the decision to place the patient on long-term anticoagulation. During subsequent clinic visits between 2014 and 2018, his INR remained within the therapeutic range of 2-3 at doses between 63 mg and 70 mg per week. By February 2018, his viral load was undetectable, and he was switched to lamivudine, tenofovir, and dolutegravir in December 2018 as part of the phasing out of old regimens. His viral load, as of June 2019, remained undetectable on the new regimen. During the next anticoagulation clinic visit, however, his INR was supratherapeutic at 6.5 without any report of bleeding. Four doses of warfarin were held, after which he continued on his normal dose. A week later, his INR was 5.5, and warfarin was subsequently held for a week. After holding warfarin, his INR dropped to 1.4, and warfarin was restarted at a dose of 56 mg per week, representing a 10% reduction in his previously stable weekly dose of 69 mg warfarin prior to starting dolutegravir. Over the next 2 weeks, his INR was 2.4 and 3.2, respectively, and he was asked to return in 1 month. After returning in 1 month, his INR had risen to 6.5 again and necessitated holding four doses of warfarin with the weekly dose being reduced to 35 mg per week. In the subsequent two weeks, his INR was 1.4 and 1.7, respectively. The weekly dose was increased to 42 mg, and a therapeutic INR of 2.3 was achieved. His INR remained therapeutic at 2.2 two weeks later, and his weekly dose of 42 mg was maintained.  Figure 1 shows the changes in INR and warfarin dose that have been described above.

### 2.2. Case 2

**Figure 2:**
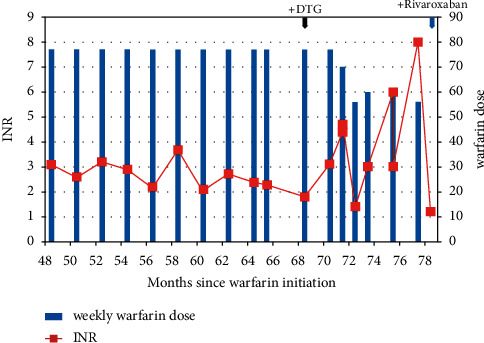
Graphical representation of case 2's INR and corresponding weekly warfarin dose during clinical visits.

Case 2 describes a 60-year-old woman diagnosed with HIV in June 2010 and started on lamivudine, nevirapine, and zidovudine. Since 2010, her CD4 count has risen from 182 cells/mm^3^ at initiation to 465 cells/mm^3^ in August 2012. In September 2011, she was diagnosed with an unprovoked lower limb DVT, with HIV as the only risk factor, and started on warfarin to achieve an INR of 2-3. A therapeutic INR of 3.3 was attained in February 2012 at a warfarin dose of 77 mg per week (11 mg once daily). A lower limb Doppler ultrasound done that month revealed a chronic DVT which resulted; hence, she was placed on extended prophylaxis. Apart from two separate instances in April 2012 and March 2013, when, due to an explained dietary variation, her INR shot up to 4.6 and 5.1, respectively, her INR has remained therapeutic or near therapeutic at doses between 69 and 77 mg. In line with guideline recommendations, she was switched to a dolutegravir-based regimen in June 2019. Her viral load at the time of the switch was 148 copies/mL and decreased to 29 copies/mL by September 2019. In the same month, her INR went up to 4.7, and her warfarin dose was reduced by approximately from 10% to 68 mg/week. INR was 4.7 two weeks later. One dose of warfarin was held, and the dose was reduced to 56 mg/week. A subsequent INR of 1.4 was observed, and the warfarin dose was increased to 60 mg/week. A month later, in November 2019, her INR was 3.0 and warfarin was maintained at the same dose. In January 2020, her INR had shot up to 6.0. Three doses of warfarin were held, and the weekly dose reduced to 56 mg. INR subsequently dropped to 3.0 two weeks later. In March 2020, her INR was found to be above 8; hence, warfarin was held for 2 weeks. INR fell to 1.2, and she was restarted on a weekly dose of 42 mg. In April 2020, due to concerns over bleeding and for her convenience, she was switched to rivaroxaban. There have not been any report of bleeding or thrombosis since switching to rivaroxaban.  Figure 2 shows the changes in INR.

## 3. Discussion

Dolutegravir is an integrase strand inhibitor with a high barrier to resistance. It has proven efficacy in treatment of naïve patients and is superior to efavirenz and darunavir/ritonavir and no inferior and more convenient, due to its one daily dosing, when compared to other integrase inhibitors such as raltegravir [12–14]. In treatment experienced patients who are virally controlled, dolutegravir was shown to be noninferior to standard therapy and superior to raltegravir [15, 16].

It is also effective in INSTI-experienced patients with resistance to raltegravir and eltegravir [17]. For these reasons, national HIV programs around the world, especially those supported by PEPFAR, have been making DTG-based regimens the preferred treatment option. This natural experiment provides us with an opportunity to better understand the potential drug interactions and adverse effects which may occur due to this transition in treatment.

Nevirapine, a CYP 3A4 and CYP 2B6 substrate, was previously part of the first-line treatment regimen for national HIV programs as seen within the two cases described here. Nevirapine is an enzyme inducer that increases its own metabolism and that of other substrates. Long-term nevirapine use may increase ALT, AST, and GGT levels [18]; however, alterations of these labs would not provide much insight on the observed interactions as changes in ALT, AST, and GGT would be nonspecific and not elucidate the extent of induction and subsequent deinductions. This phenomenon was observed in rat models where induction of the CYP 3A and CYP2B enzymes was not associated with nevirapine hepatotoxicity [19].

Our patients did not exhibit any signs of liver dysfunction; hence, no liver function tests were conducted. Monitoring of asymptomatic laboratory abnormalities is not routinely done in our setting and thus not included within this case report. Routine monitoring of transaminase levels is typically conducted during the initiation of nevirapine therapy to identify acute hepatotoxicity but has not been shown to be useful in identifying induction [20]. Magnitude of microsomal enzyme induction may be quantified more accurately using mRNA expression and enzyme assays which are not available in our setting and typically require cost prohibitive specialized laboratory instrumentation [21, 22].

Warfarin, an anticoagulant used in the management of VTEs, is also metabolized by CYP3A4 and CYP 2C9. Its levels will therefore be reduced when used together with nevirapine, necessitating the use of higher doses to achieve therapeutic INR, as observed in our patient. However, upon stopping nevirapine, the metabolism of warfarin reduces, leading to an elevation in warfarin blood levels and INR that may warrant a reduction in warfarin dose [10]. Enzyme induction involves signaling for and de novo synthesis of new enzymes; hence, the process of deinduction will be determined by cessation of signaling and degradation of enzymes. It may take up to 3 weeks for nevirapine levels to become undetectable and several weeks for deinduction to occur.

Furthermore, the CYP2B6 variant of slow metabolizers occurs more in people of African descent and results in lengthening of the nevirapine half-life, which may prolong the induction of enzymes after nevirapine is stopped [23–25].

The expected pharmacokinetics of the alteration in warfarin disposition following a switch from a nevirapine-based regimen to a dolutegravir-based regimen highlight the importance of close monitoring of the INR for several weeks after the switch is made. In both cases, the time course for the reaction took several weeks as the deinduction process associated with nevirapine led to a dangerous supratherapeutic INR for both patients. Clinicians must be aware of the unique pharmacokinetics of this common antiretroviral medication switch to avoid the increased risk of bleeding that patients may be exposed to without timely alteration of warfarin dosing.

## 4. Conclusion

Clinicians caring for patients on warfarin who are switched from a nevirapine-based regimen to a dolutegravir-based regimen should provide close INR monitoring of patients for several weeks after halting nevirapine. This can best be achieved by ensuring that the care of patients at the HIV clinic and anticoagulation clinic is integrated to ensure that efforts to monitor this potentially dangerous interaction are coordinated.

## Data Availability

The data used to support the findings of this study are included within the article.
